# Epidemiology and Elimination of HCV-Related Liver Disease

**DOI:** 10.3390/v10100545

**Published:** 2018-10-06

**Authors:** Pierre Pradat, Victor Virlogeux, Eric Trépo

**Affiliations:** 1 Centre for Clinical Research, Groupement Hospitalier Nord, Hospices Civils de Lyon, 69004 Lyon, France; pierre.pradat@univ-lyon1.fr; 2 Université Claude Bernard Lyon 1, INSERM 1052, CNRS 5286, Centre Léon Bérard, Centre de Recherche en Cancérologie de Lyon, 69008 Lyon, France; victor.virlogeux@gmail.com; 3 Lyon University, Lyon, France; 4 Department of Hepatology, Croix-Rousse Hospital, Hospices Civils de Lyon, 69004 Lyon, France; 5 Liver Unit, Department of Gastroenterology and Hepatopancreatology and Digestive Oncology, CUB Hôpital Erasme, Université Libre de Bruxelles, 1070 Bruxelles, Belgium; 6 Laboratory of Experimental Gastroenterology, Université Libre de Bruxelles, 1070 Bruxelles, Belgium

**Keywords:** hepatitis C virus, direct-acting antiviral agents, hepatocellular carcinoma, cancer recurrence, cirrhosis, sustained virological response

## Abstract

Hepatitis C virus (HCV) infection, defined by active carriage of HCV RNA, affects nearly 1.0% of the worldwide population. The main risk factors include unsafe injection drug use and iatrogenic infections. Chronic HCV infection can promote liver damage, cirrhosis and hepatocellular carcinoma (HCC) in affected individuals. The advent of new second-generation, direct-acting antiviral (DAA) agents allow a virological cure in more than 90% of treated patients, and therefore prevent HCV-related complications. Recently, concerns have been raised regarding the safety of DAA-regimens in cirrhotic patients with respect to the occurrence and the recurrence of HCC. Here, we review the current available data on HCV epidemiology, the beneficial effects of therapy, and discuss the recent controversy with respect to the potential link with liver cancer. We also highlight the challenges that have to be overcome to achieve the ambitious World Health Organization objective of HCV eradication by 2030.

## 1. Introduction

The treatment of chronic hepatitis C virus (HCV) infection has undergone a recent revolution since the advent of new second-generation, direct-acting antiviral (DAA) agents with initial marketing authorization obtained in 2013 and 2014 in most Western countries. These new, very effective molecules left behind the triple therapy comprising an NS3/4A protease inhibitor that arrived in 2011 and the pegylated-interferon/ribavirin combination (PEG-IFN/RBV), a dual therapy that has been used for more than 15 years. These new treatments allow the long-term viral eradication and therefore a virological cure in more than 90% of treated patients. This remarkable efficiency is, however, associated with a high financial cost, having initially led to a treatment restriction to patients with advanced fibrosis or with particular comorbidities subject to prioritization. In all Western countries, the decrease in costs associated with an increase in the number of molecules on the market will lead to the universal access to HCV treatment very soon, making it possible to cover all patient populations.

## 2. Epidemiology of HCV

Chronic HCV infection, defined by active carriage of HCV RNA in the blood, is a major public health problem worldwide, affecting more than 71 million people and responsible for 400,000 deaths per year [1]. About 1.75 million new cases were estimated worldwide in 2015 [2].

### 2.1. Prevalence of HCV Infection

#### 2.1.1. Prevalence in the General Population and Distribution Worldwide

A recent study, supported by the World Health Organization (WHO) Global Hepatitis Report 2017, estimated the number of viraemic infections at 71.1 million (95% confidence interval (95% CI): 62.5–79.4) in the world in 2015, representing a prevalence of 1.0% (95% CI: 0.8–1.1) [1]. Using a different methodology, this study provides an estimate well below the 130 to 150 million chronically infected individuals previously reported [3]. This study is based on a review of the literature of HCV epidemiological studies published between January 2000 and March 2016 and carried out in 110 different countries (representing 92% of the world population). To estimate the prevalence of HCV in these 110 countries in 2015, the authors use, for the first time, a Markov model applied to each country. This model is able to consider the temporal factor and thus makes it possible to extrapolate the prevalence rates reported previously to 2015. To support this new approach, a Delphi method (which relies on consultation of local experts) was used to validate the input data of the model in each of the considered countries. In total, the prevalence could be estimated for 59 countries. For the remaining 41 countries with a lack of sufficiently robust data, the literature data had to be used directly to establish a reliable model. This novel method thus makes it possible to renew the conservative method which could lead to overestimations in the past. However, this study was able to consider only 110 out of the 250 countries recognized worldwide, due, on the one hand, to a lack of data from the literature and, on the other hand, to the quality of the source data, which varies greatly from country to country. This study is currently the only one to consider the temporal factor and, in particular, the impact of new treatments on the epidemiology of HCV and can therefore serve as a reference.

Using the results published in this latest study, the prevalence of HCV RNA varies greatly by region of the world. The prevalence is particularly high in Central Asia and Eastern Europe, affecting more than 3% of the population in these regions. Central Europe, North Africa, Central Africa, West Africa, the Middle East and Australasia are areas where the prevalence of HCV RNA is 3%, reaching less than 1% in other regions of the world, including Western European countries (the average prevalence being 0.5% in the latter). Migrants from Asia, sub-Saharan Africa and Eastern Europe contribute to an increase in prevalence in the receiving countries of Western Europe and North America [4].

Regarding the number of cases, a large proportion of infected people live in South and East Asia with 25.8 million estimated cases, 8.5 million live in North Africa/Middle East, 4.7 million in Southeast Asia and 2.3 million in Western Europe. The five countries with the highest number of individuals chronically infected with HCV are in descending order: China (9.8 million), Pakistan (7.1 million), India (6.2 million), Egypt (5.6 million) and Russia (4.7 million).

#### 2.1.2. Prevalence of HCV Infection in Different Risk Groups

Among intravenous drug users (IVDU), the overall HCV seroprevalence was estimated at 52.3% (95% CI: 42.4–62.1) in the world in 2011, using data from 77 different countries [5]. However, HCV seroprevalence varied greatly from region to region with midpoints reports ranging from 9.8% to 97%. The three countries with the largest numbers of IVDU, i.e., China, Russia and the USA, had midpoint estimates of HCV seroprevalence of 67.0%, 72.5% and 73.4% respectively. After extrapolating the data to all countries, the authors estimated that in 2010, about 10 million IVDU (range: 6–15.2 million) were anti-HCV positive worldwide.

Few studies have been published on the worldwide seroprevalence of HIV-HCV coinfection compared to HCV monoinfection. The last major epidemiological study of coinfection was a meta-analysis based on 783 epidemiological studies reporting seroprevalence data from HIV-HCV coinfection between January 2002 and January 2015, with a minimum population of 50 individuals [6]. The total number of coinfected individuals was estimated at 2.3 million (interquartile (IQ): 1.3–4.3 million), half of whom being IVDU (1.2 million, IQ: 0.89–1.4 million). The overall prevalence of HCV-infected individuals among persons living with HIV (PLWH) was estimated at 2.4% (IQ: 0.8–5.8). The prevalence was 4.0% (IQ: 1.2–8.4) within pregnant or heterosexually exposed samples, 6.4% (IQ: 3.2–10.0) in men having sex with men (MSM) and 82.4% (IQ: 55.2–88.5) in IVDU. Among the regions most affected by co-infection, the study mentioned Eastern Europe and Central Asia with 607,700 individuals, sub-Saharan Africa with 429,600 individuals followed by North America with 319,000 people. In Western Europe a total of 103,800 co-infected individuals were estimated. This meta-analysis, however, was representative of only 88 countries in the world, with no data available in the 106 other countries identified during the review process. In addition, the authors again emphasized the great heterogeneity of data quality according to the considered countries. This study also estimated the seroprevalence of co-infection and not the prevalence of active HCV infections in PLWH because of the limited available data on the prevalence of HCV RNA (only 10% of the studies included in the meta-analysis provided data on the prevalence of HCV RNA).

HCV infection is also more frequent in prisoners than in the general population. The last available data regarding HCV prevalence in this population was published in 2013 [7]. Based on 93 sources of data for anti-HCV prevalence among general detainees, the authors estimated an overall prevalence of 26% (95% CI: 23–29%) in their meta-analysis, despite a high heterogeneity I^2^ of 100%. This high heterogeneity rate was due to large variations between samples regarding history of injecting drug use and to the year of data collection, as more recent sources tend to have lower anti-HCV prevalence. Moreover, wide variations of seroprevalence was observed by geographical region with the lowest estimation in the Middle East and North Africa (3%; 95% CI: 2–5%) and the highest in Central Asia (38%, 95% CI: 32–43%).

### 2.2. Incidence of HCV Infection

#### 2.2.1. HCV Incidence in the General Population

Incidence data are rarely available in the literature. Several studies estimated the HCV incidence using a mathematical back-calculation method as part of a static model representing the natural history of HCV [8,9,10,11]. To estimate the incidence of HCV infection, the authors used the total number of HCV infections occurring a given year and subtracted the number of spontaneous clearances, the number of people cured and the number of deaths. Overall, the incidence rate has already peaked in most countries, except for Russia where the rate continues to rise [9]. This peak incidence was observed in 1970 in Egypt, Sweden and Mongolia; in the early 1980s in New Zealand, Norway, Saudi Arabia and Canada; between 1989 and 1992 for the European countries and the United States, while for Finland, Iran, Australia, Brazil and the Czech Republic, the peak was observed more recently in the late 1990s until the early 2000s [8,9,10,11]. In Pakistan, the incidence peaked and then plateaued in 2005 [10].

#### 2.2.2. HCV Incidence in Different Risk Groups

Regarding the IVDU population, a meta-analysis based on 16 studies reporting HCV incidence in both female- and male-IVDU reported an overall pooled HCV incidence of 20.4/100 person-years (PY) in females (95% CI (13.9–29.9)) vs 15.2/100 PY in males (95% CI (10.5–22.0)) [12]. The highest incidence rates were reported from China, with 76.3/100 PY in females and 29.8/100 PY in males in a prospective cohort study following 333 HIV-seronegative IVDU for 36 months from 2002 [13]. The authors also reported a significant decrease of HCV incidence during the three years of follow-up, decreasing from 43.9 per 100 person-years in 2002 to 15.6 per 100 person-years in 2004. A French study, based on the combination of a regression model and a deterministic compartmental model, reported much lower incidence rates and showed that the incidence of HCV infection decreased from 7.9/100 PY in 2004 to 4.4/100 PY in 2011, suggesting the effectiveness of prevention measures such as needle exchange programs in this at-risk population [14].

Few studies have reported data on incidence rates of HCV infection in the HIV population. A prospective cohort study that followed 1941 PLWH in nine different centers in the United States between 2000 and 2013 reported an overall incidence rate of 1.07/100 PY, with an overall decrease over the 13 years [15]. The incidence decreased indeed from 1.83/100 PY in 2000–2003 to 0.94/100 PY in 2004–2007, then stabilized at 0.95/100 PY in 2008–2010 before declining to 0.88/100 PY in 2011–2013. Among the risk groups included in this cohort (61% of MSM, 30% of heterosexuals, 3% of IVDU and 6% of other/unknown), the incidence rate was 3.4% PY for IVDUs, 1.04% PY for MSM, 0.92% PY for heterosexuals and 0.97% PY for the other/unknown risk group. Over the study period, a decrease in the incidence rate was observed among IVDU and heterosexuals, but not among MSM.

Numerous studies have focused more specifically on the HIV-positive population, and a meta-analysis based on 17 studies that followed a total of 13,000 HIV-positive MSM between 1984 and 2012 reported an increase in HCV incidence from 0.42% PY in 1991 to 1.09% PY in 2010 and 1.34% PY in 2012, with, among seroconverters, a very high proportion reporting high-risk sexual practices [16].

Several recent studies reported similar results in the MSM population, with an increasing incidence rate in recent years in relation to high-risk sexual practices [17,18,19,20,21] and HCV transmission clusters have even been identified in series of cases of acute hepatitis C [22,23].

A French modeling study based on the large Dat’AIDS cohort from 2012 to 2016 explored the dynamics of infection in different at-risk groups: MSM, heterosexuals, IVDU, and other at-risk groups, and assessed the potential impact of DAA treatment over the next 10 years on the epidemiology of HIV-HCV co-infection [24]. The authors showed that high HCV treatment uptake will result in a dramatic decline of active HCV infection within the next 10 years in French PLWH from all risk groups, except in high-risk MSM in which high-risk sexual practices, such as unprotected anal intercourse, fisting, group sex, or the use of nasal or intravenous drugs during sex, contribute to the still on-going HCV spread in this population [19,25,26,27].

### 2.3. HCV Screening Recommendations

A major issue to stem the HCV outbreak is to identify all HCV active carriers in each country by strengthening routine screening. However, such a screening is complicated since it is estimated that 90% of HCV infected people in the world ignore their infection [28,29]. The last available recommendations from WHO for HCV testing have been published in 2016 and recommended that HCV serology testing should be offered to individuals from three different populations: (i) Individuals from the most affected population, i.e., who are part of a population with high HCV seroprevalence or who have a history of HCV risk exposure/behavior; (ii) all individuals from the general population if HCV antibody seroprevalence is ≥2% or 5% and (iii) specific identified birth cohorts of older persons at higher risk of infection and morbidity within populations that have an overall lower general prevalence [30]. On the other hand, some countries have already implemented well-defined screening guidelines: In the United States, for instance, screening is strongly recommended for all those born during the “baby boom” period, i.e., from 1945 to 1965. It was indeed estimated that 73% of the US population infected with HCV was born during this period [31]. In Canada, systematic screening of the cohort of subjects born between 1945 and 1975 is also recommended. At the EU level, current screening recommendations advocate the early identification of chronically-infected pregnant women, implementation or strengthening of testing activities for high-risk groups, as well as free-of-charge liver enzymes testing in routine medical check-ups [32]. Recently developed Rapid Diagnostic Tests (RDTs) represent a promising new tool for broadscale screening of HCV infection in high- to medium-risk populations [33]. These tests facilitate screening by targeting previously hard-to-reach populations.

## 3. Beneficial Effects of HCV Treatment

IFNα-2b monotherapy in 1991 was the first treatment approved by the Food & Drug Administration (FDA) to treat chronic hepatitis C (CHC) [34]. This initial therapy allowed a modest 15–20% of sustained virological response (SVR) and induced significant side effects including asthenia, neutropenia/thrombocytopenia, myalgia and influenza-like syndrome [35,36]. In 1998, SVR rates were increased to 38% in patients treated with the combination of IFNα-2b with daily oral administration of ribavirin (RBV) [37]. In 2001 and 2002, the FDA approved Pegylated-IFN (PEG-IFN)α-2b and PEG-IFNα-2a respectively, which allowed IFN-based therapy to be administered on a weekly basis. The combination of PEG-IFNα-2a or PEG-IFNα-2b and ribavirin allowed SVR in 56% and 54% of treated patients [38,39]. This regimen given for 6 to 12 months depending on the HCV genotype became the new standard of care [34]. After ten years of stagnation with PEG-IFN/RBV combination, a recent and exemplary revolution spread over several waves. The first one was observed in 2011 with the launch of the first DAAs. More than 75% of patients infected with HCV genotype 1 treated with the NS3/4A protease inhibitors (telaprevir and boceprevir) in combination with PEG-IFNα achieved an SVR; however, both had clinically relevant side effects and increased the daily pill burden for patients [40,41]. These were rapidly followed by the second wave from 2014 of all oral regimens (i.e., IFN-free) based on anti-NS5A (e.g., daclatasvir, ledipasvir) and NS5B (e.g., sofosbuvir) polymerase inhibitors and 2nd generation protease inhibitors (e.g., grazoprevir**)** increasing SVR rates to 90-98% in naïve and PEG-IFN/RBV-experienced patients [42,43,44,45]. Finally, a third wave recently emerged with the introduction of the so-called pangenotypic DAAs able to treat the whole HCV population [46,47,48,49].

### 3.1. Impact of Treatment Response on Liver Fibrosis, Function, and Extrahepatic Manifestations

An SVR following treatment is currently defined by the absence of viral RNA in the blood 12 weeks after stopping treatment [50]. In the majority of patients (57–94%) SVR after IFN-based therapy allows a decrease in inflammation and an improvement in the fibrosis score, although in a minority of patients (1–14%), fibrosis continues to progress despite virological cure [51]. The regression of fibrosis after SVR was significantly associated with age and platelet count. The presence of cofactors, such as alcohol intoxication or a metabolic syndrome may, however, influence the expected beneficial effect of treatment on fibrosis. Thanks to the implementation of DAAs, even in the most advanced forms of cirrhosis, viral clearance has been shown to improve liver function at 6 months compared to untreated patients [52]. SVR has also been associated with a reduction in portal hypertension and a reduction of the risk of liver decompensation [53]. Finally, SVR after both IFN-based and IFN-free therapies has been linked to the reduction of HCV extrahepatic manifestations, such as cryoglobulinemia vasculitis, renal impairment, insulin resistance and type 2 diabetes, compared to non-SVR patients as shown by a recent meta-analysis summarizing 48 studies [54].

### 3.2. Impact of HCV Treatment on the Risk of Hepatocellular Carcinoma (HCC)

#### 3.2.1. De novo HCC

Patients treated with IFN-based therapy achieving SVR have been shown to have a significantly lower risk of developing HCC than those who did not [55,56,57,58,59]. It was therefore expected that DAAs introduction would have an even more stringing impact on HCC rate reduction. Surprisingly, an unexpectedly high HCC incidence in cirrhotic patients under and following DAA regimens was reported by several teams [60,61]. Underlying hypothetical biological mechanisms speculated that a brutal interruption of HCV viral load would result in a drastic immunologic and molecular modification in the liver microenvironment and favor liver carcinogenesis [62,63]. However, these observations came from retrospective, mostly single-center case series of initial DAAs implementation with limited sample sizes. Conversely, analysis from registries restricted to patients treated with DAAs did not report a higher HCC risk compared with patients treated with IFN [64,65,66,67]. These much larger cohort studies had also methodological limitations, including their retrospective design [64,65,66], the lack of unequivocally confirmation of cirrhosis by liver biopsy [64,65,66], usage of coding system for the diagnosis of cirrhosis and HCC [65,66], exclusion of HCC patients during early follow-up [65], or the lack of detailed information concerning the liver cancer surveillance program [65,66]. A meta-analysis gathering 26 studies on HCC occurrence between January 2000 and February 2017 concluded that there was no evidence for differential HCC occurrence risk following SVR from DAA and IFN-based therapy [68].

Recently, the RESIST-HCV study, a multicenter prospective cohort including 2249 patients with HCV-cirrhosis treated with DAA from Sicily, Italy [69], highlighted the importance of cirrhosis stage in HCC occurrence. After one year of therapy the incidence of HCC was significantly lower in patients with SVR compared to those without SVR in Child Pugh A and Child Pugh B patients (2.1% vs. 6.6%, *p* < 0.001 and 7.8% vs 12.4% *p* < 0.001 respectively). Overall, the incidence of HCC in patients with SVR was 3.7-fold higher in Child Pugh B patients than in those with compensated cirrhosis (2.1% vs. 7.8% *p* < 0.001). Univariate analysis of the CirVir prospective cohort, including 1270 patients with compensated biopsy proven HCV-cirrhosis from 35 French centers, revealed a significantly higher 3-year cumulative incidence of HCC in the DAA group compared to the SVR-IFN group (5.9% vs. 3.1% *p* = 0.03) [70]. Nevertheless, it is without surprise that patients in the DAA group included a much higher proportion of advanced cirrhotic stages than in the SVR-IFN group, since IFN-regimen have always been recommended in fully compensated patients. Moreover, patients were older in the DAA group and displayed more comorbidities (e.g., type 2 diabetes). After adjustment for these well-known confounders [71], HCC incidence was similar, regardless of treatment allocation [70]. Thus, inclusion of patients with more advanced cirrhosis-stage compared to those in clinical trials or from historical cohorts of patients treated with IFN-based therapy, and failure to account for confounding factors, might explain results from initial alarming retrospective studies. Nahon et al. [70], also suggested that patients in the DAA group might have been followed more irregularly and that the quality of imaging may have been reduced. As a result, they speculated that DAA may have been initiated in patients with pre-existing undetectable oncogenic lesions, increasing the risk of de novo HCC during early follow-up. This hypothesis was also underlined by the analysis of a large Italian registry [72]. The main limitation of current prospective studies remains their short follow-up in DAA-treated patients, and long-term observation will be required [69,70].

Some studies also raised that HCCs developed after DAA therapy displayed a more aggressive pattern with more multifocal and more severe tumor stages [72,73]. Nevertheless, this finding has not been observed in other large cohort studies [66,69,70], and might be explained by the same aforementioned reasons.

#### 3.2.2. Risk of HCC Recurrence

HCC recurrence after tumor resection is a frequent complication with a 5-year incidence of 70% [74]. A meta-analysis gathering 10 cohorts concluded that IFN-based regimen could lead to a 74% reduction of HCC recurrence with a higher benefit in patients with viral clearance, but this study had methodological limitations such as population heterogeneity or inclusion of non-randomized studies with possible incomplete data collection and selection bias [75,76]. The use of DAAs led to more conflicting results (Table 1). First, in a multicenter cohort of 58 patients from Spain, Reig et al. reported an abnormally high recurrence rate of 27.6% for a median follow-up of 5.7 months [77]. SVR rate was 98% at the time of analysis and 90% of patients received a curative treatment option for their HCC. Similarly, an Italian single cohort study also reported an HCC recurrence rate of 28.8% in 59 patients over a median follow-up period of 6 months. SVR rate after DAA treatment was 90%, and 80% of patients received a curative treatment option for their previous HCC. In multivariate logistic regression analysis exploring the factors associated with HCC recurrence, a lower age and an advanced fibrosis score were significantly associated with HCC recurrence [60]. In the two studies, the proportion of recurrence was much higher than the 7.4% (range 0–12.5%) 6-month recurrence rate reported by a meta-analysis evaluating untreated HCV patients in this clinical context [78]. By contrast, analysis of three large French prospective cohorts (France Recherche Nord & sud Sida-vih Hépatites HEPATHER, CirVir and CUPILT) did not report a significant difference between DAA-treated and untreated patients [79].

Since these early studies, many cohort studies have been conducted to analyze this issue (Table 1). Of the 14 published studies available as of 1 June 2018, four concluded that there was an increased risk of HCC recurrence after DAA treatment [60,77,80,81] while the other 10 did not conclude in an increased risk, the recurrence rate being compared or not to a control group according to the study design [79,82,83,84,85,86,87,88,89,90].

Among these identified studies, much heterogeneity should be stressed regarding the methodology employed:

- Half of the studies had no control group: as a result, they had to compare their reported HCC recurrence rate with the data from the literature;

- the number of included patients in the different groups was low most of the time with only four studies presenting results for more than 100 patients in one of the groups;

- HCC treatment options varied substantially between studies: some considered patients treated with curative therapeutic options only while others considered both curative and palliative options (i.e., transarterial chemoembolization and percutaneous alcoholization);

- the start date of the follow-up period varied mainly between the beginning of DAA treatment and the date of HCC remission;

- the duration of follow-up strongly varied according to the studies ranging from a few months to several years;

- the design was retrospective for 11 of them: to compensate for the lack of randomization in this type of design, four studies used a propensity score either by matching exposed and unexposed patients together or by inverse probability weighting;

- the type of statistical analyses: some used the reference method for this type of study, i.e., survival analysis, and explored the potential factors associated with HCC recurrence thanks to multivariate analyses while others only estimated a crude rate of HCC recurrence over the follow-up period.

Other factors not reported in Table 1, but that could have contributed to the heterogeneity regarding the methodology are: (i) The frequency and type of HCC recurrence follow-up imaging; (ii) the almost systematic lack of distinction between patients with a single history of HCC and those with multiple histories of HCC before being treated with DAA; (iii) the heterogenous delay between HCC remission date and the start of DAA treatment and (iv) the type of recurrence, i.e., local or distant, single, multiple or infiltrating nodules. These different methodological aspects and the great variability observed in the different studies can partly explain the heterogenous conclusions reported. For the moment, there are no more convincing studies than others, as all of them have more or less important methodological weaknesses.

On the other hand, a meta-analysis evaluating 17 studies did not observe a significant difference in HCC recurrence between DAA 12.2/100 PY (95% CI: 5.00–29.6) and IFN-based therapy 9.21/100 PY (95% CI: 7.18–11.8) [68]. Another meta-analysis based on 24 studies evaluating HCC recurrence after DAA treatment concluded that HCC recurrence rate was acceptable after DAA treatment more particularly if DAA therapy was delayed at least 6 months after HCC complete response [91].

As already mentioned in the case of de novo HCC, the inclusion of patients with advanced cirrhosis stage who were excluded from previous IFN-based regimen, and thus with potential undetectable neoplastic lesions by current imaging methods, may explain these unexpected high, early recurrence-rates in some retrospective studies who failed to fully account for confounding factors [75]. On the other hand, the studies reporting higher HCC recurrence rate after DAA treatment suggest again a hypothetical mechanism implying an impaired anti-tumor response following a sudden decrease of HCV replication [92,93].

In conclusion, HCV viral clearance with DAAs lowers the risk of HCC occurrence even in advanced cases of cirrhosis [69]. However, the risk of HCC development remains even after SVR [57], and justifies long-term follow-up in cirrhotic patients [94]. Owing to their immunomodulating influence and therefore potential impact on liver carcinogenesis, DAA-based therapy should only be initiated in HCC naïve patients after exclusion of (pre)neoplastic lesions [70]. A follow-up period without treatment after liver resection to rule out cancer cell disseminations should also be recommended. Long-term follow-up of existing prospective studies and new ones will better define individual treatment strategies.

### 3.3. Impact of HCV Treatment on the Epidemiology of HCV

Since the arrival of the new DAAs on the market with very high SVR rates, the question of the possible eradication of HCV has emerged, at least in countries where treatments are available on a large scale.

#### 3.3.1. The WHO Program

Thanks to DAA treatments that brought optimism about potential eradication of HCV, WHO launched a program in May 2016 to “eradicate” viral hepatitis by the year 2030 [95]. Unfortunately, this objective was too ambitious and WHO has therefore defined these eradication goals for 2030 as: (i) 90% reduction in the incidence of new HCV infections; (ii) 100% HCV screening on blood donation; (iii) 90% of injections made with sterile or disposable equipment; (iv) 300 sterile syringes per year for each IVDU via exchange programs; (v) detection of 90% of patients chronically infected with HCV; (vi) treatment of 80% of patients chronically infected with HCV.

The objectives of WHO in the general population have been evaluated both in developed countries where access to DAA treatment is simplified and prevention of HCV transmission is developed, and in developing countries. In these studies, the authors demonstrate the significant difference between the different countries considered, with the need to “only” increase the treatment rate in European countries to achieve the goals while in developing countries, the objectives in terms of screening and treatment rates for 2030 are unrealistic [96,97,98].

To eliminate HCV infection and try to achieve the goals set by the WHO in 2030, several issues remain to be taken into account with among them: (i) the rate of people diagnosed is still too low because of the “silent” aspect of the infection especially in the early stages of the disease; (ii) difficult access to DAA treatments and screening tools at reasonable costs in all countries; (iii) restricted access to DAA in some countries according to fibrosis stage, genotype, certain comorbidities, and IV drug use; (iv) the lack of interventions to reduce the risk of transmission in at-risk populations such as IVDU and MSM; (v) high reinfection rates in the MSM population, with possible transmission between HIV(−) and HIV(+), in particular through the introduction of pre-exposure prophylaxis (PreP) programs and high-risk practices; (vi) the existence of viral strains resistant to DAA treatments; (vii) the existence of international HCV transmission networks among HIV(+) MSM.

These different issues are the issues to be resolved through a joint effort of public health authorities at the local, national and international levels, but also of all the actors involved in the healthcare system including pharmaceutical companies.

#### 3.3.2. HIV-HCV Coinfected Population

One of the patient populations infected with HCV for which monitoring data are more readily available is the HIV-HCV co-infected population. Using data from the French Dat’AIDS cohort, we found that the prevalence of active HCV infection among the HIV population drastically decreased since the arrival of the DAAs, to reach 5.4% at the end of 2015. Despite these positive results, several studies in various countries pointed out the recent increase in the incidence of HCV infection in MSM with high-risk practices. Following this observation, the question of “total” eradication of HCV infection was therefore potentially jeopardized.

We recently showed that model projections made in each of the risk groups over the next 10 years have a similar impact with a drastic decrease in the prevalence of co-infection, except for MSM with high risk practices [24]. In this subgroup, considering an annual treatment initiation rate of 30%, the prevalence of co-infection is expected to decrease from 7% in 2016 to 6.3% in 2026, but the total number of individuals should increase from 700 to more than 800 individuals. However, an annual treatment rate of 50% and 70% would reduce the prevalence in this subgroup at 2.4% and 1.3%, respectively.

## 4. Conclusions

Currently, the race to “perfectovir” is coming to an end. A short treatment duration (8–24 weeks) with the last available DAAs allow SVR in more than 95% of cases. These DAAs are very well tolerated and accessible to both the least severe patients with minimal fibrosis and have been shown to be beneficial even in the most advanced form of cirrhosis [52]. The presence of comorbidities is no longer a barrier, and the choice of a specific molecule is based on potential drug interactions and renal function. However, 5% of treated patients still develop treatment failure either following relapse at the end of treatment or by the selection of resistance mutations mainly in the NS5A protein [99]. For these patients, rescue therapies are necessary with possibly longer treatment duration (24 weeks) and addition of RBV [100].

Data from large registries [64,65,66], and prospective cohorts [69,70] in cirrhotic patients treated with DAA do not support an increased risk of de novo HCC. On the contrary, viral clearance has been associated with a significantly lower risk of HCC occurrence. Similarly, meta-analyses and prospective studies have shown no evidence for a significantly increased risk of HCC recurrence [68,79]. Alarming results from retrospective studies [60,61,77], might result from a selection bias and inclusion of a higher proportion of patients with decompensated cirrhosis and comorbidities [69,70,75]. However, DAA immunomodulating effect might have a potential influence on liver carcinogenesis, and should still warn caution. Thus, DAA-based therapy should be initiated after ruling out the presence of a concomitant neoplastic lesion or tumor cell dissemination after resection. Given both the exceptional efficacy and tolerability of DAAs, the design of a randomized control trial including an untreated group would be unethical. Long-term follow-up of existing prospective studies and new ones will better define future individual treatment strategies.

The 2016 WHO objective of “eradicating” HCV as a public health problem in 2030 is an ambitious target: In 2015, estimates showed that only 20% of HCV-infected patients were diagnosed and only 7% had started treatment, with very large country-dependent variations [101]. One of the main barriers to treatment access is their elevated cost, prompting many countries to establish access restrictions. However, the arrival of low-cost generic drugs is promising in resource-limited countries [102,103].

The results of modeling studies published so far are encouraging in some countries, especially if preventive measures in terms of annual treatment rates and intervention are reached [98,104,105,106,107].

## Figures and Tables

**Table 1:** Summary of published cohort studies evaluating hepatocellular carcinoma (HCC) recurrence rate after direct-acting antiviral (DAA) treatment as of 1st June, 2018.

Authors	Sample Size	Start Date of Follow-Up (D0)	Curative Option for HCC Treatment ^1^	HCC Recurrence Rate	Length of Follow-Up	Design/Statistical Analyses	SVR	Conclusions
**Increased HCC Recurrence Rate**								
El Kassas et al. (2018) [81]	● 53 DAA treated patients ● 63 non-treated	Start of DAA treatment	100%	● DAA: 4.1% PM ● non-treated: 1.0% PM	● DAA: 16 months ● non-treated: 23 months	● Prospective ● Poisson regression and propensity score (inverse probability weighting)	77%	● Increased HCC recurrence rate
Conti et al. (2016) [60]	● 59 DAA treated patients	Start of DAA treatment	81%	Crude rate: 29%	24 months	● Retrospective ● No control group ● Risk factors analysis	91%	● Increased HCC recurrence rate
Reig et al. (2016) [77]	● 58 DAA treated patients	Start of DAA treatment	90%	Crude rate: 28%	5.7 months	● Retrospective ● No control group	98%	● Increased HCC recurrence rate
Bielen et al. (2017) [80]	● 41 DAA treated patients	Start of DAA treatment	98%	Crude rate: 15%	6 months	● Retrospective ● No control group	94%	● Potential increase of HCC recurrence rate
**No Increased Recurrence Rate**								
The ANRS collaborative study group on hepatocellular carcinoma. (2016) [79]	● HEPATHER cohort: - 189 DAA treated patients - 78 non-treated ● CirVir cohort: 79 incidental HCC treated by percutaneous ablation or liver resection - 13 DAA treated patients - 66 non-DAA treated ● CUPILT cohort 314 liver transplant recipients for HCC and treated with DAA	Date of inclusion in the cohort	ND	● HEPATHER - DAA: 0.73% PM - Non-treated: 0.66% PM ● CirVir - DAA: 1.11/100 PM - No DAA: 1.73/100 PM ● CUPILT Recurrence rate of 2.2%	● HEPATHER - DAA: 20 months (from the start of DAA treatment) - Non-treated: 26 months (from D0) ● CirVir 21.3 months ● CUPILT 70.3 months	● Retrospective analysis of prospective cohorts ● Multivariate analysis	● HEPATHER 92% ● CirVir 100% ● CUPILT 97%	● No increased of HCC recurrence rate between DAA treated and non-treated patients
Ikeda et al. (2017) [86]	● 89 DAA treated patients ● 89 non-treated (matched)	HCC remission date	77%	At 1 year: ● DAA: 18% ● control: 22% At 2 years: ● DAA: 25% ● control: 47%	● DAA: 1.8 year ● Control: 5.3 years	● Retrospective ● Propensity score (matching) ● Survival analysis	90%	● Decreased HCC recurrence rate
Cabibbo et al. (2017) [83]	● 143 DAA treated patients	Start of DAA treatment	82%	● 6 months: 12% ● 12 months: 27% ● 18 months: 29%	8.7 months	● Prospective ● No control group ● Multivariate survival analysis	96%	● No increased HCC recurrence rate
Ogawa et al. (2018) [88]	● 152 DAA treated patients	HCC remission date	85%	At 1 year: ● Non-cirrhotic: 7% ● Cirrhotic: 23%	NA	● Retrospective ● No control group ● Multivariate survival analysis	100%	● No increased HCC recurrence rate
Nagata et al. (2017) [90]	● 83 DAA treated patients ● 60 PR ± PI treated patients	HCC remission date	100%	At 5 years: ● DAA: 54% ● PR ± PI: 45%	● DAA: 2.3 years ● PR ± PI: 6.2 years	● Retrospective ● Propensity score (matching) ● Multivariate survival analysis	DAA: 96% PR ± PI: 65%	● No difference between the two groups
Adhoute et al. (2018) [87]	● 22 DAA treated patients ● 49 non-treated (matched)	HCC diagnosis date	63%	Crude rate: ● DAA: 41% ● Control: 35%	● DAA: 68 months ● Non-treated: 32 months	● Retrospective ● Propensity score (matching) ● Univariate survival analysis	86%	● No difference between the two groups
Warzyszyńska et al. (2017) [85]	● 19 DAA treated patients ● 32 non-treated	HCC remission date	100%	At 1 year: ● DAA: 53% ● Non-treated: 25%	NA	● Retrospective ● Univariate survival analysis	95%	● No difference between the two groups
Zavaglia et al. (2017) [84]	● 31 DAA treated patients	Start of DAA treatment	100%	Crude rate: 3.2%	8 months	● Retrospective ● No control group	96%	● No increased HCC recurrence rate
Mashiba et al. (2018) [89]	● 78 IFN or PR or PR + PI treated patients ● 347 DAA treated patients	End of DAA treatment	ND	● No statistical difference between the two groups (crude rate NA)	● Minimum: 24 months after antiviral treatment	● Retrospective	100%	● No difference between the two groups
Virlogeux et al. (2017) [82]	● 23 DAA treated patients ● 45 non-treated	HCC remission date	79%	● DAA: 1.7/100 PM ● Non-treated: 4.2/100 PM	● DAA: 36 months ● Non-treated: 15 months	● Retrospective ● Multivariate survival analysis ● Propensity score (covariate)	96%	● Decreased HCC recurrence rate

^1^ Non curative HCC treatment options: Transarterial chemoembolization and alcoholization; PR: PEG-interferon/Ribavirin; IFN: PEG-interferon; NA: Not available; PM: Person-month; PI: NS3/4A protease inhibitor.
